# 2′-^19^F labelling of ribose in RNAs: a tool to analyse RNA/protein interactions by NMR in physiological conditions

**DOI:** 10.3389/fmolb.2024.1325041

**Published:** 2024-02-14

**Authors:** Hesna Kara, Alexander Axer, Frederick W. Muskett, Carlos J. Bueno-Alejo, Vasileios Paschalis, Andrea Taladriz-Sender, Sumera Tubasum, Marina Santana Vega, Zhengyun Zhao, Alasdair W. Clark, Andrew J. Hudson, Ian C. Eperon, Glenn A. Burley, Cyril Dominguez

**Affiliations:** ^1^ Department of Molecular and Cellular Biology, Henry Wellcome Building, University of Leicester, Leicester, United Kingdom; ^2^ Leicester Institute of Structural and Chemical Biology, Henry Wellcome Building, University of Leicester, Leicester, United Kingdom; ^3^ WestCHEM and Department of Pure and Applied Chemistry, University of Strathclyde, Glasgow, United Kingdom; ^4^ School of Chemistry, University of Leicester, Leicester, United Kingdom; ^5^ Biomedical Engineering Research Division, School of Engineering, University of Glasgow, Glasgow, United Kingdom

**Keywords:** RNA-protein interaction, ^19^F NMR spectroscopy, RNA binding proteins, RNA labelling, concurrent/competitive binding

## Abstract

Protein-RNA interactions are central to numerous cellular processes. In this work, we present an easy and straightforward NMR-based approach to determine the RNA binding site of RNA binding proteins and to evaluate the binding of pairs of proteins to a single-stranded RNA (ssRNA) under physiological conditions, in this case in nuclear extracts. By incorporation of a ^19^F atom on the ribose of different nucleotides along the ssRNA sequence, we show that, upon addition of an RNA binding protein, the intensity of the ^19^F NMR signal changes when the ^19^F atom is located near the protein binding site. Furthermore, we show that the addition of pairs of proteins to a ssRNA containing two ^19^F atoms at two different locations informs on their concurrent binding or competition. We demonstrate that such studies can be done in a nuclear extract that mimics the physiological environment in which these protein-ssRNA interactions occur. Finally, we demonstrate that a trifluoromethoxy group (-OCF_3_) incorporated in the 2′ribose position of ssRNA sequences increases the sensitivity of the NMR signal, leading to decreased measurement times, and reduces the issue of RNA degradation in cellular extracts.

## 1 Introduction

The interaction between RNAs and RNA-binding proteins (RBPs) is an essential, fundamental, and highly regulated cellular process. A large number of proteins regulate post-transcriptional gene expression, such as splicing and translation (Baltz et al., 2012); indeed, there are approximately 800 RBPs in humans (Castello et al., 2012). Modulation of their interaction with RNA or changes in their levels of expression can lead to neurodegenerative diseases and cancers (Cieply and Carstens, 2015; Kelaini et al., 2021; Prashad and Gopal, 2021; Mehta et al., 2022; Sanya et al., 2023). Many RBPs act on pre-mRNA splicing to regulate the production of alternative isoforms of mRNA, leading to the production of different proteins with different functions or to the production of isoforms with poison exons (exons containing premature termination codons) that downregulate protein expression (Jobbins et al., 2023; Manabile et al., 2023; Nikom and Zheng, 2023) These RBPs interact specifically with their pre-mRNA targets through various RNA binding domains (RBDs) such as an RNA Recognition Motif (RRM) (Maris et al., 2005; Daubner et al., 2013) or a KH domain (Valverde et al., 2008; Nicastro et al., 2015). These small domains generally only recognize specifically 3 to 5 nucleotides of a single-stranded RNA (ssRNA) with moderate affinities (dissociation constants in the micromolar range) and the high affinity and specificity of RBPs towards RNA comes from the fact that most splicing factors contain multiple RBDs, each binding a different region of the same RNA. Pre-mRNAs often contain binding sites for many different RBPs in or near their exons and the cooperative or competitive binding of these RBPs to the ssRNA is believed to ultimately dictate the outcome of alternative splicing events (Nag et al., 2022).

NMR spectroscopy is a powerful tool to study protein-RNA interactions at the atomic level (Pellecchia, 2005; Dominguez et al., 2011; Furukawa et al., 2016; Teilum et al., 2017). However, NMR studies of macromolecules requires the labelling of one macromolecule with an “NMR-visible” isotope. While ^13^C and ^15^N isotope labelling of proteins is routine, this precludes the possibility of following the binding at the level of the RNA. This is especially problematic if there is more than one binding site for the protein on the RNA, or if there is any intention to follow the binding of two or more proteins to the same RNA molecule. Labelling of RNA with ^13^C and/or ^15^N is less straightforward and requires either complex chemical synthesis or *in vitro* transcription of the RNA using expensive ^13^C-labelled nucleotides (Nikonowicz et al., 1992; Batey et al., 1995; Wenter et al., 2006). Furthermore, because the ^1^H chemical shift distribution of atoms in ssRNA is narrow, their specific assignment is difficult due to signal overlap and therefore NMR spectroscopy studies of ssRNAs are generally limited to short oligonucleotides.

Some of these limitations can be avoided by the use of fluorine atoms at specific sites within biomolecules, such as RNA (Gronenborn, 2022)**.** The natural isotope of fluorine, ^19^F, has a 100% natural abundance, possesses a spin of ½, has a high NMR sensitivity and covers a much wider chemical shift range than ^1^H (Dolbier, 2009). Like ^1^H, the chemical environment of a ^19^F atom strongly affects its chemical shift. This has allowed the use of ^19^F NMR spectroscopy to investigate RNA secondary structures and protein conformations (Kreutz et al., 2005; Sharaf and Gronenborn, 2015; Sharaf et al., 2016; Overbeck et al., 2023). Although fluorine atoms are not naturally present in most macromolecules such as proteins and nucleic acids, they can be incorporated at specific positions in nucleotides and amino acids by chemical modifications (Kreutz et al., 2005; Scott and Hennig, 2016). Structural investigations of RNAs using ^19^F NMR have been performed by incorporating ^19^F atoms at position 4′of the ribose of uridines (Li et al., 2020), at position 5 on the bases of cytidines and uridine (Becette et al., 2020; Nußbaumer et al., 2020; Sudakov et al., 2023), at position 2 of adenine (Scott et al., 2004), at position 2′on the ribose of any nucleotide (Kreutz et al., 2005; Fauster et al., 2012; Himmelstoss et al., 2020), or at the 5′end of the RNA by adding a 3,5-bis(trifluoromethyl)phenyl/Benzyl group (Bao and Xu, 2018, 2020). Protein-RNA interactions have also been investigated by ^19^F NMR: while most studies relied on incorporation of fluorinated amino acids into proteins (Campos-Olivas et al., 2002; Cellitti et al., 2008; Crowley et al., 2012; Sharaf and Gronenborn, 2015; Matei and Gronenborn, 2016; Sharaf et al., 2016; Dujardin et al., 2018; Gronenborn, 2022), in some cases, fluorine was incorporated into RNAs to investigate their interaction with proteins or small molecules (Fauster et al., 2012; Himmelstoss et al., 2020; Gronenborn, 2022), or to monitor the modification of an RNA by an enzyme (Li et al., 2020). In these cases, it was shown that protein binding induced either a chemical shift change, or a broadening of the ^19^F signal. However, these studies were done on structured RNAs, not ssRNAs, and the position of the ^19^F atom was chosen mainly to investigate the structure of the RNA rather than the interaction with proteins.

All the NMR studies mentioned above were done *in vitro* in NMR suitable buffers. However, NMR studies in cellular extracts (bacterial, *xenopus* oocytes, mammalian) have demonstrated that it is also possible to obtain structural information on macromolecules, such as proteins and nucleic acids, in physiological conditions and they are easier to conduct than in-cell NMR (Luchinat and Banci, 2022; Theillet and Luchinat, 2022). For example, NMR studies of nucleic acids in cell extract has been carried out to monitor the maturation of tRNAs in yeast extract (Barraud et al., 2019) or to analyse ssDNA/protein interaction by ^19^F labelling of the protein and NMR analysis in *E. coli* cell lysate (Welte et al., 2021).

Here we demonstrate that ^19^F labelling of a ssRNA at different specific positions allows for the mapping of different RBP binding sites and can inform on the concurrent or competitive binding of pairs of RBPs to the ssRNA, using ^19^F NMR spectroscopy in the presence of mammalian cells nuclear extracts. This involved the incorporation of 2′-F or 2′-OCF_3_ nucleotides at specific positions within a portion of the SMN2 pre-mRNA and measuring 1D ^19^F NMR spectra in HeLa nuclear extract, in the absence or presence of individual or pairs of three RBPs, Sam68, hnRNP A1 and SRSF1.

## 2 Materials and methods

### 2.1 Overexpression and purification of the proteins

The DNA sequence coding for Sam68 QUA1KH (Uniprot: Q07666, amino acids 96–260) and hnRNP A1 RRM12 (Uniprot: P09651, amino acids 1–218) were cloned in the pLEICS-01 vector (https://le.ac.uk/mcb/facilities-and-technologies/protex/) that contains the sequence encoding for a N-terminal hexa-Histine tag followed by a TEV protease site and a gene for Ampicillin resistance. The DNA sequence coding for SRSF1 RRM12 (Uniprot: Q07955, amino acids 1–196 with Y37S and Y72S mutations in RRM1) was cloned in the plasmid pET24b that contains the sequence encoding for a N-terminal GB1-His-TEV tag.

All proteins were purified as described previously (Barraud and Allain, 2013; Cléry et al., 2013; Feracci et al., 2016). In brief, plasmid DNAs were transformed into Rosetta BL21 DE3 cells and the protein expression was induced by the addition of 0.5 mM (Sam68 and hnRNP A1) or 1 mM (SRSF1) of isopropylthiogalactosidase (IPTG) for 16 h at 25°C. Proteins were purified by affinity chromatography using Ni-NTA agarose (Quiagen) and dialysed overnight in the presence of TEV protease. SRSF1 was separated from the GB1-His-TEV tag by NI-NTA affinity chromatography. Sam68 and hnRNP A1 were further purified by gel filtration using a Superdex 75 column (GE Healthcare). Sam68, SRSF1 and hnRNP A1 were dialysed against the NMR buffer (20 mM NaHPO_4_ pH 6.5, 100 mM NaCl, 1 mM beta-mercaptoethanol, 50 mM L-Arginine, 50 mM L-Glutamate). Protein concentration was estimated by measuring the absorbance at 280 nm and using theoretical molar absorption coefficients of 7450 M^-1^ cm^-1^ for Sam68, 13,075 M^-1^ cm^-1^ for A1 and 22,015 M^-1^ cm^-1^ for SRSF1.

### 2.2 Chemical synthesis of fluorinated RNAs

2′Fluoro (2′-F) RNAs:

The 2′-F RNAs were chemically synthesized by Dharmacon (Horizon Discovery) with one fluorine modification in RNA1 and two in RNA2:

RNA1: UUACA-(2′-F-G)GGUUUUAGACAAAAU.

RNA2: UUACA-(2′-F-G)GGUUUUAGACAAA-(2′-F-A)U.

Fluorine was inserted in the 2′position on the ribose of G6 of RNA1 and G6 and A20 of RNA2.

2′-*O*-Trifluoromethylated (2′-OCF_3_) RNA:

The 2′-OCF3 adenosine phosphoramadite was chemically synthesized following a previously published protocol (Himmelstoss et al., 2020) (see Supplementary Material S1). This phosphoramadite was then incorporated by chemical synthesis (Horizon Discovery) at position A20 in RNA3:

RNA3: UUACAGGGUUUUAGACAAA-(2′-OCF3-A)U.

### 2.3 Nuclear extract

Nuclear Extract from HeLa cells was purchased from Ipracell (catalogue number: CC-01–20–005).

### 2.4 NMR sample preparation

Lyophilised RNAs received from Dharmacon were dissolved in 20 μL of NMR buffer and heated to 95°C for 5 min then snap cooled to 4°C in ice. RNA concentrations were estimated by measuring the absorbance at 260 nm using an extinction coefficient of 224,300 M^-1^ cm^-1^ for 2′-F-RNAs and 229,000 M^-1^ cm^-1^ for 2′-OCF_3_-RNA.

For the RNA only samples, the RNAs were diluted to a final concentration of 100 or 200 μM in 600 µL NMR buffer containing 10% D_2_O. For RNA/protein samples, the RNAs were mixed with Sam68 QUA1-KH, hnRNPA1 RRM12 or SRSF1 RRM12, 10% D_2_O and buffer to a final volume of 600 µL with final concentrations of 200 µM for the RNA and 400 µM for the proteins (2′-F-RNA experiments) or 100 µM of RNA and 200 µM of protein (2′-OCF_3_-RNA experiments).

### 2.5 NMR experiments

600 µL of RNA or RNA/protein mixture were inserted into 5 mm NMR tubes. All NMR spectra were recorded at 303 K (typical temperature used for *in vitro* splicing assays) on a Bruker AVIII-600 MHz spectrometer equipped with a 5 mm HF SEF probe with z-gradients. 1D ^19^F spectra of the 2′-F-RNAs samples were acquired with a spectral width of 20 ppm, a frequency offset of −202 ppm, and a total acquisition time of 100 ms. The total number of scans was 3,840 (1.5 h per experiment). 1D ^19^F spectra of the 2′-OCF_3_ RNA samples were acquired with a spectral width of 20 ppm, a frequency offset of −60 ppm, and a total acquisition time of 100 ms. The number of scans was 1,024 for the comparison between 2′-F-RNA2 and 2′-OCF_3_ RNA3 (29 min per experiment) and 256 for the experiment with 2′-OCF_3_ RNA, 40% nuclear extract in the absence or presence of Sam68 QUA1-KH (7 min per experiment). NMR data were processed and analysed using TOPSPIN 3.6 (Bruker). All the experiments were zero-filled to 8,192 points, and a 6 Hz exponential window function applied.

### 2.6 Electromobility shift assays (EMSA)

EMSA assays were done using 14% polyacrylamide (40% (w/v)29:1 Acrylamide:Bis-Acrylamide) gels in TBE buffer (Tris 0.13 M, Borate 45 mM, EDTA 2.5 mM pH 7.6 and run in TBE buffer at 4° at 160 V for 1 h, using Xylene cyanol as loading dye. Gels were stained with toluidine blue for 5 min, then rinsed with water overnight on a gel rotator.

#### 2.6.1 Binding of Sam68 to fluorinated RNAs *versus* unmodified RNA

All the samples contained 2 μL of unmodified RNA2 or 2′F-RNA2 or 2′-OCF3-RNA3 at a final concentration of 40 μM. Then, 10 μL of NMR buffer for the free RNA sample or 10 μL of protein in NMR buffer were added to obtain different final concentrations of Sam68: 10 μM, 50 μM, 100 μM or 200 μM. Finally, 3 μL of Xylene Cyanol non denaturing loading dye was added to each sample.

#### 2.6.2 Competition assay for SRSF1 and Sam68 for binding to 2′F-RNA

The RNA concentration was 40 μM in all samples and the proteins concentrations (Sam68 or SRSF1) were 80 μM. Samples in nuclear extract contained the same concentrations of RNA and proteins with the addition of 40% HeLa nuclear extract.

## 3 Results

**FIGURE 1:**
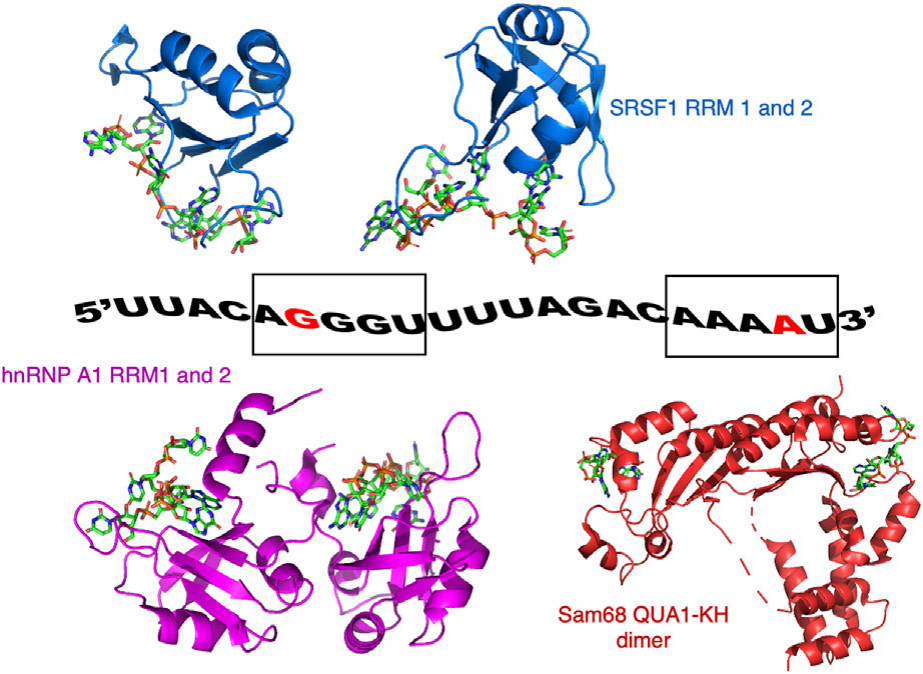
Model of ssRNA/protein binding used in this study. The RNA corresponds to a portion of the SMN2 pre-mRNA. The guanine at position 6 (G6) and the adenine at position 20 (A20) that were ^19^F labelled on their 2′ribose position are coloured red. G6 is part of the AGGGUU motif that binds SRSF1 and hnRNP A1, while A20 is part of the AAAAU motif binding Sam68. Also shown are the structures of SRSF1 RRM1 and RRM2 bound to RNA (pdb codes: 6HPJ and 2M8D), hnRNP A1 RRM1 and RRM2 bound to RNA (pdb codes: 5MPG and 5MPL), and a structural model of Sam68 bound to RNA based on the structure of the homologous protein T-STAR (pdb code: 5ELT).

In our study, we have used a 21-nucleotide portion of SMN2 pre-mRNA (encompassing the 3′end of intron 6 and the 5′end of exon 7) and the RBDs of three proteins: Sam68 QUA1-KH domain, SRSF1 RRM1-2 domains and hnRNP A1 RRM1-2 domains (Figure 1). Based on biochemical data (Wee et al., 2014) and structural work (Barraud and Allain, 2013; Cléry et al., 2013; Feracci et al., 2016; Beusch et al., 2017), this RNA contains binding sites for SRSF1 (GG (A/G)), hnRNP A1 ((U/C)AGG) and Sam68 ((A/U)AA (Figure 1). The SRSF1 and hnRNP A1 binding sites overlap near the 5′end of the RNA, while the Sam68 binding site is located near the 3′end (Figure 1).

2′fluorinated (2′-F) nucleotides were used as these nucleotides are commercially available and do not affect RNA thermodynamic stability or structure (Kreutz et al., 2005). Furthermore, the 2′position is rarely involved in ssRNA-protein complex formation (Dominguez et al., 2011; Corley et al., 2020), and 2′-F nucleotides are sensitive to conformational changes in RNA structures and protein binding (Kreutz et al., 2005). Thus, we hypothesized that ^19^F labelling of the ribose 2′ position would provide a useful tool for investigating the simultaneous binding profile of several RBPs in physiological conditions. To confirm that the 2′-F modification does not significantly interfere with the binding of RBPs, we tested the binding of Sam68 with the unmodified and 2′-F-modified RNA using electromobility shift assay (EMSA) (Supplementary Figure S1). As expected, Sam68 binds both RNAs at the concentrations used for the NMR studies.

**FIGURE 2:**
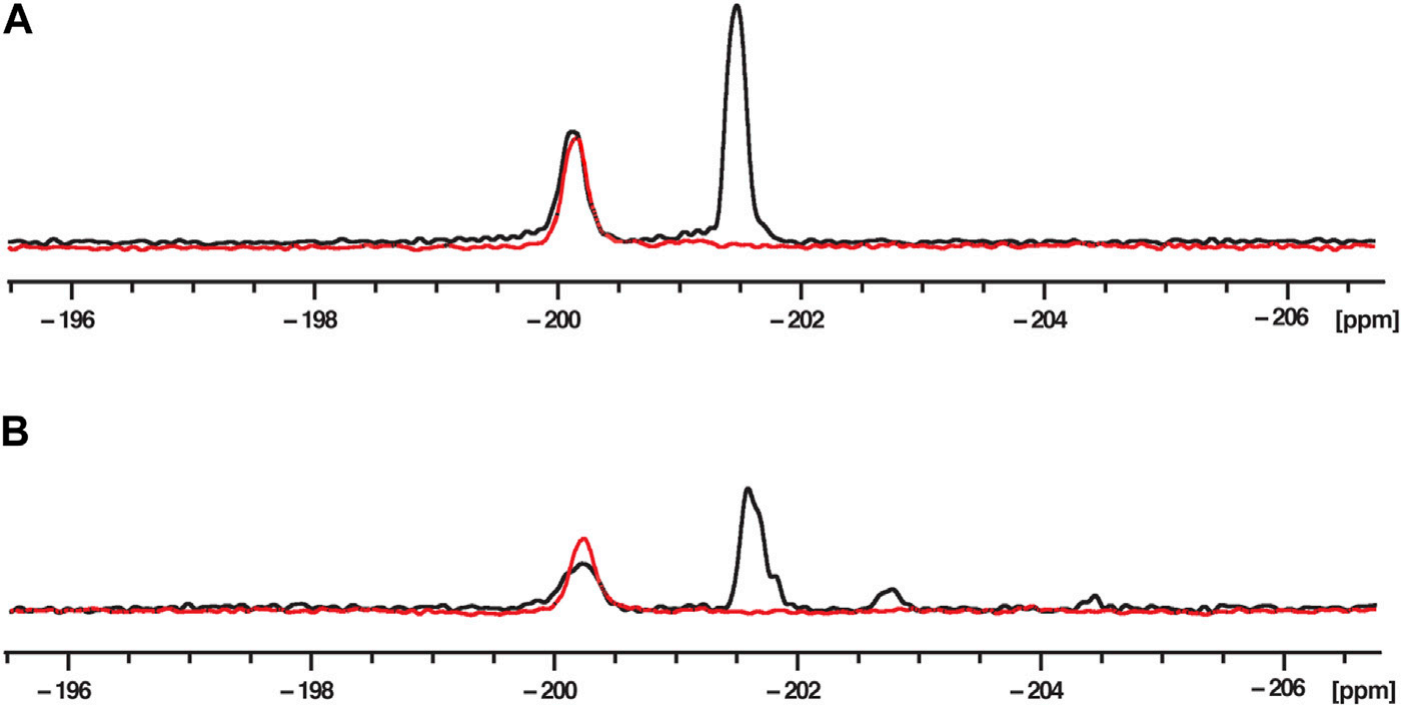
^19^F NMR spectra at 303K of fluorinated RNAs in phosphate buffer **(A)** and nuclear extract **(B)**. RNA2 containing two 2′-F at G6 and A20 (UUACA-(2′-F-g)GGUUUUAGACAAA-(2′-F-A)U) is in black and RNA1 containing only one 2′-F at G6 (UUACA-(2′-F-g)GGUUUUAGACAAAAU) is in red. The RNA concentration was 200 μM and the total NMR measurement time per spectrum was 1.5 h (3,840 scans).

To assign the chemical shifts of the ^19^F NMR signals, two 2′-F RNA sequences were used. The first RNA (RNA1) contained a single 2′-F nucleotide at position 6 (G6) in the middle of the hnRNP A1 and SRSF1 binding sites, while the second RNA (RNA2) contained two 2′-F nucleotides at positions G6 and at position 20 (A20), in the Sam68 binding site (Figure 1). 1D ^19^F NMR spectra of these RNAs were recorded in the absence of proteins in buffer (Figure 2A). The ^19^F NMR spectrum of RNA2 containing two 2′-F nucleotides (G6 and A20) shows two distinct peaks, one at −200.2 ppm and one at −201.5 ppm (black), while the spectrum of RNA1 containing a single 2′-F nucleotide (G6), displays a single peak at −200.2 ppm (red). We conclude that the peak at −200.2 ppm corresponds to the G6 ^19^F signal and the peak at −201.5 ppm corresponds to the A20 ^19^F signal. This indicates that the ^19^F signal at position 2′of the ribose is highly sensitive to the nature of the base and/or to its local environment in the RNA, and hence allows the differentiation of the two positions in the ssRNA.

To mimic the physiological conditions of the RNA-RBP interactions, ^19^F 1D NMR spectra were acquired in the presence of 40% HeLa nuclear extract (Figure 2B), because this reflects the amount of nuclear extract typically used to recapitulate splicing events in *in vitro* splicing assays (Higgins and Hames, 1994; Eperon et al., 2000). The spectra showed signals at the same chemical shifts as those recorded in phosphate buffer, as we observe two peaks at −200.2 and −201.5 ppm for RNA2, and only one peak (−200.2 ppm) for RNA1. While the peaks observed in nuclear extract are slightly broader than those in buffer, they are clearly visible and suitable to investigate the effect of protein addition on these signals. Minor peaks are also observed between −202 and −206 ppm and we suspected that they arise from the partial degradation of the RNA due to the presence of RNAses in the nuclear extract. We therefore incubated RNA2 in 40% nuclear extracts and measured 1D ^19^F spectra as a function of time (Supplementary Figure S2). As expected, the signals of G6 and A20 disappear with time (after 10 and 6 h, respectively, which is consistent with A20 being located one nucleotide from the 3′end of the RNA while G6 is located 6 nucleotides from the 5′end), confirming that the peaks observed between −202 and −206 ppm arise from RNA degradation in the nuclear extract. Indeed, we can speculate that the new peak at −202.7 ppm corresponds to a partial degradation of the 3′end of the RNA because this peak appears almost immediately after addition of nuclear extract (within 10 min) and seems to correlate with the rapid disappearance of the A20 signal. The degradation peak at −204.7 ppm could be due to 5′end degradation because it appears later (after about 1 h) and correlates with the disappearance of the G6 peak. We could then assume that the peak at −203.2 ppm that appears much later (after 2 h) corresponds to the signals of both G6 and A20 when the RNA is fully degraded. In that case, the 2′-F of both G6 and A20 experience the same chemical environment and therefore have the same chemical shift.

It is therefore important to measure the NMR spectra immediately after the addition of the nuclear extract to the RNA. In conclusion, our data demonstrate that it is possible to observe and differentiate two ^19^F signals located in two different positions of the same ssRNA even in the presence of nuclear extracts.

**FIGURE 3:**
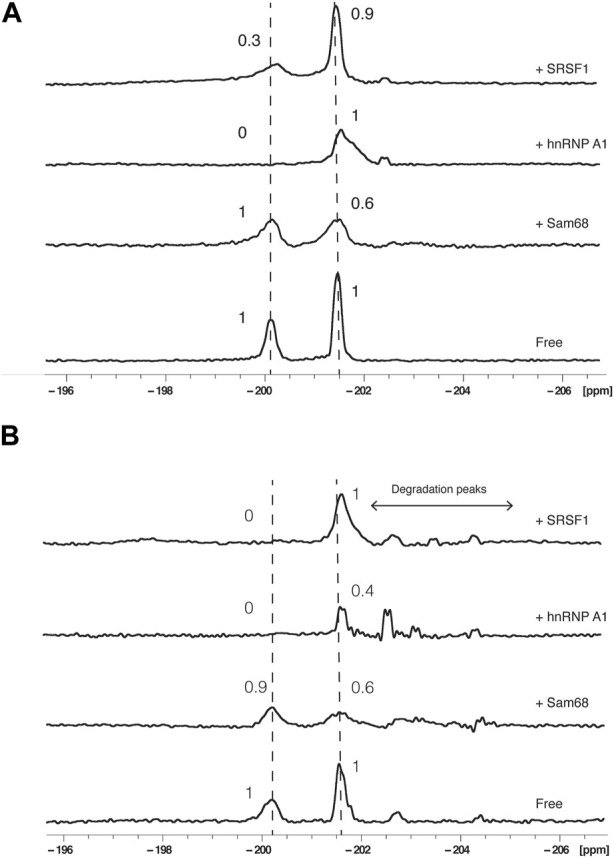
Effect of proteins on ^19^F signals of fluorinated RNA. ^19^F NMR spectra at 303K of RNA2 in the absence or presence of Sam68, hnRNP A1 or SRSF1 RNA binding domains in the absence **(A)** or presence of 40% nuclear extract **(B)**. The total NMR measurement time per spectrum was 1.5 h (3,840 scans). Peak area ratios are indicated.

Next, the RBDs of Sam68, SRSF1 or hnRNP A1 were added to RNA2 in molar excess (RNA:protein molar ratio of 1:2) and ^19^F NMR spectra were recorded either in buffer (Figure 3A) or in presence of 40% nuclear extract (Figure 3B). Addition of the proteins did not lead to obvious chemical shift changes but induced a decrease and a broadening of the signals. We therefore analysed our data by integrating the relative peak area of each signal and calculating the ratios of these integrals before and after the addition of the proteins using the formula:

$$\text{Peak integral ratio}\left(\text{PIR}\right)=\left(I/I_{0}\right)$$

Where I is the peak integral in the presence of protein and I_0_ is the peak integral in the absence of protein.

The results were similar in either buffer (Figure 3A) or in the presence of 40% nuclear extract (Figure 3B): Addition of Sam68 did not alter the signal of G6 (PIR of 1 and 0.9 in buffer and nuclear extract, respectively) but induced a reduction in the PIR of A20 (0.6 in both conditions), consistent with Sam68 only interacting with the motif AAAAU (Feracci et al., 2016). In contrast, the addition of SRSF1 affected the signal of G6 (PIR of 0.3 and 0) but not A20 (PIR of 0.9 and 1), consistent with SRSF1 only interacting with the UACAGGGU motif (Cléry et al., 2013). Addition of hnRNP A1 strongly affected the signal of G6 (PIR of 0 in both conditions) but also induced a broadening but no reduction in peak integral (PIR of 1) of the A20 signal in buffer conditions, while in the presence of nuclear extract, the A20 signal intensity is reduced (PIR of 0.4). This suggest that hnRNP A1 binds predominantly the CAGGGU motif as expected (Barraud and Allain, 2013; Beusch et al., 2017), but the binding also slightly influences the chemical environment of A20, possibly through weaker binding near this motif as suggested previously (Kashima and Manley, 2003; Cartegni et al., 2006). In the presence of nuclear extracts, we also observe minor peaks between −202 and −205 ppm resulting from the partial degradation of the RNA in the nuclear extract. These peaks are also observed following the addition of the proteins (notably hnRNP A1 and SRSF1) and could be due to trace amount of RNAses present in the protein samples after purification. It is worth noting that the degradation peaks observed are more prominent upon addition of hnRNP A1 or SRSF1 than addition of Sam68. This is consistent with the fact that SRSF1 and hnRNP A1 bind near the 5′end of the RNA, leaving the 3′end accessible to RNAses, while Sam68 binding near the 3′end protects the RNA from 3′end degradation. The fact that the major degradation peak observed in these experiments is at −202.7 ppm is also consistent with our previous assumption that this peak is associated with 3′end degradation of the RNA.

Taken together, these data demonstrate that addition of RBPs affect predominantly the NMR signal located near the protein binding site (G6 for hnRNP A1 and SRSF1, and A20 for Sam68) indicating that it is possible to identify the RNA motif bound by a protein using ^19^F NMR, even in the presence of nuclear extracts.

**FIGURE 4:**
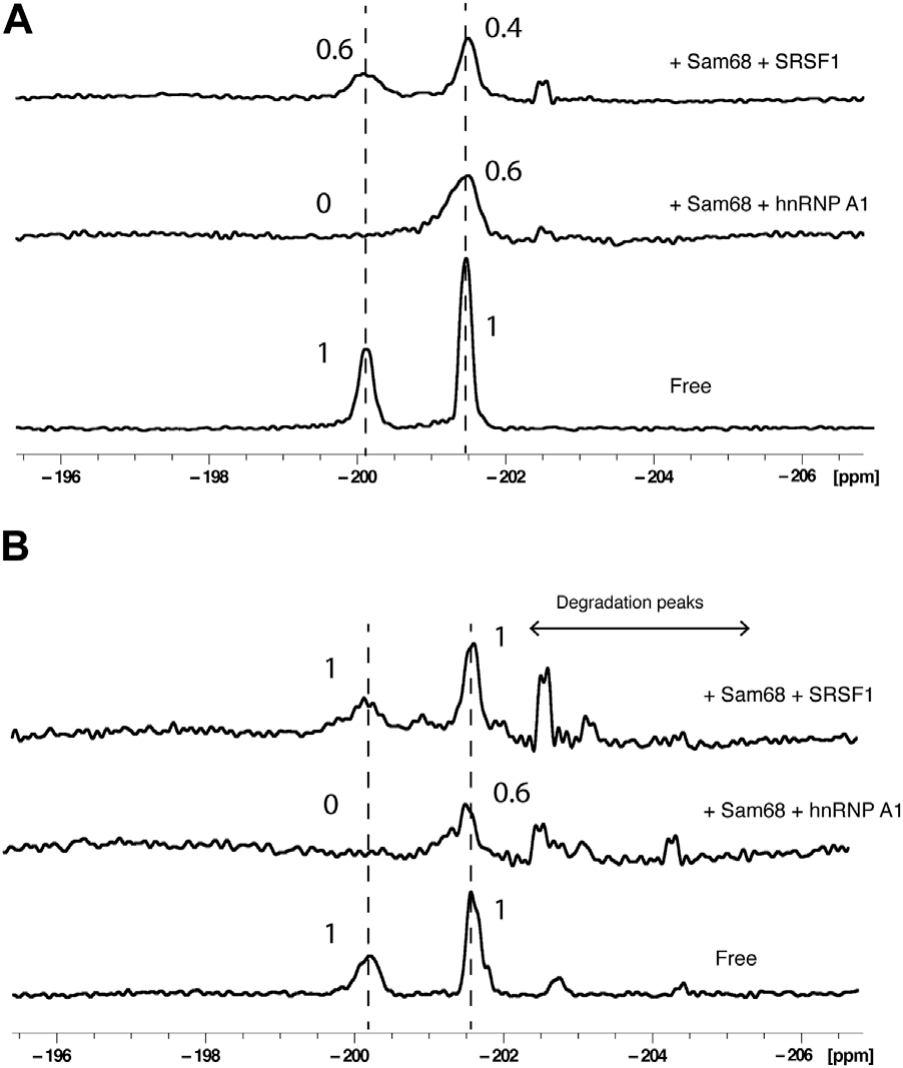
Effect of pairs of proteins on ^19^F signals of fluorinated RNA. ^19^F NMR spectra at 303K of RNA2 in the absence or presence of pairs of proteins (Sam68 + hnRNP A1 or Sam68 + SRSF1) in the absence **(A)** or presence of 40% nuclear extract **(B)**. The total NMR measurement time per spectrum was 1.5 h (3,840 scans). Peak area ratios are indicated.

Next, we added pairs of proteins to RNA2 at a RNA:protein:protein molar ratio of 1:2:2 and acquired 1D ^19^F spectra either in the absence (Figure 4A) or presence of 40% nuclear extract (Figure 4B). The addition of Sam68 and hnRNP A1 led to changes in the PIR of both peaks, very similar to the effect of Sam68 alone on the A20 signal (PIR of 0.6) or the effect of hnRNP A1 alone on the G6 signal (PIR of 0). This demonstrates that both proteins can bind concurrently to the RNA, both in buffer and in nuclear extract. In contrast, the addition of Sam68 and SRSF1 led to a different outcome. In buffer, the PIR for the A20 signal was 0.4, slightly lower than the PIR observed with Sam68 alone (0.6) indicating that Sam68 is binding the RNA in the presence of SRSF1. However, the PIR for the G6 signal was 0.6, which is higher than the PIR observed with SRSF1 alone (0.3), suggesting that SRSF1 binding might be weakened by the presence of Sam68. In nuclear extract the effect is more pronounced and the signals of G6 and A20 display a PIR of 1, indicating that when added together, Sam68 and SRSF1 do not bind, or bind weakly to the RNA. This suggests that these two RBP domains compete for binding the RNA, leading to a reduction in their binding affinity and ultimately their displacement from the RNA in the presence of other RNA binding proteins that are present in the nuclear extract. To confirm these results, we tested the binding of Sam68, SRSF1 or an equimolar amount of Sam68 and SRSF1 in buffer and in nuclear extract by EMSA (Supplementary Figure S3). In buffer, Sam68, SRSF1 and the Sam68/SRSF1 pair bind the RNA, although in the case of the Sam68/SRFS1 pair, it is not possible to determine whether both proteins or only one of them binds the RNA, since the bands corresponding to the RNA/Sam68 and RNA/SRSF1 complex overlap. In contrast, in nuclear extract, it is clear that while Sam68 and SRSF1 bind the RNA, the RNA remains free when both proteins are added together. These results are in agreement with our ^19^F NMR data.

Taken collectively, these data demonstrate that labelling a single RNA with 2′-F at two different positions is an effective tool to investigate the concurrent or competitive binding of pairs of RBPs on a single ssRNA by ^19^F NMR spectroscopy, and that the presence of nuclear extract influences the binding of pairs of proteins.

Our results above demonstrate that ^19^F NMR allows to investigate the binding of RBDs to a ssRNA in physiological conditions. However, RNA degradation is an issue and requires rapid sample preparation and acquisition time. One way of reducing acquisition time is to increase the concentration of the RNA and protein. However, fluorinated RNAs are costly, and many RBPs have a tendency to precipitate at higher concentrations. Recently, it was shown that 2′-O-trifluoromethylated (2′-OCF3) RNAs have the advantage of significant enhanced signal to noise compared to 2′-F due to the increased number of chemically equivalent ^19^F atoms and the absence of ^1^H-^19^F J-couplings (Himmelstoss et al., 2020) (Supplementary Figure S4). The synthesis of 2′-OCF3 phosphoramadites has previously been described for adenosine and cytosine (Himmelstoss et al., 2020), and more recently for guanosine and uridine (Eichler et al., 2023).

We therefore synthesized a 2′-OCF3 adenosine phosphoramidite (for details, see Supplementary Material S1) and incorporated it at position 20 of the ssRNA: UUACAGGGUUUUAGACAAA-(2′-OCF3-A)U (RNA3). To confirm that the 2′-OCF3 modification does not significantly interfere with the binding of RBPs, we tested the binding of Sam68 with the unmodified and 2′-OCF3-modified RNA using electromobility shift assay (EMSA) (Supplementary Figure S5). While the binding of Sam68 to the 2′-OCF3 RNA appears to be weaker than with the unmodified RNA, probably due to the bulkiness of the 2′-OCF3 compared to 2′-F, the binding still occurs at the concentrations used for the NMR studies (200 μM of protein).

**FIGURE 5:**
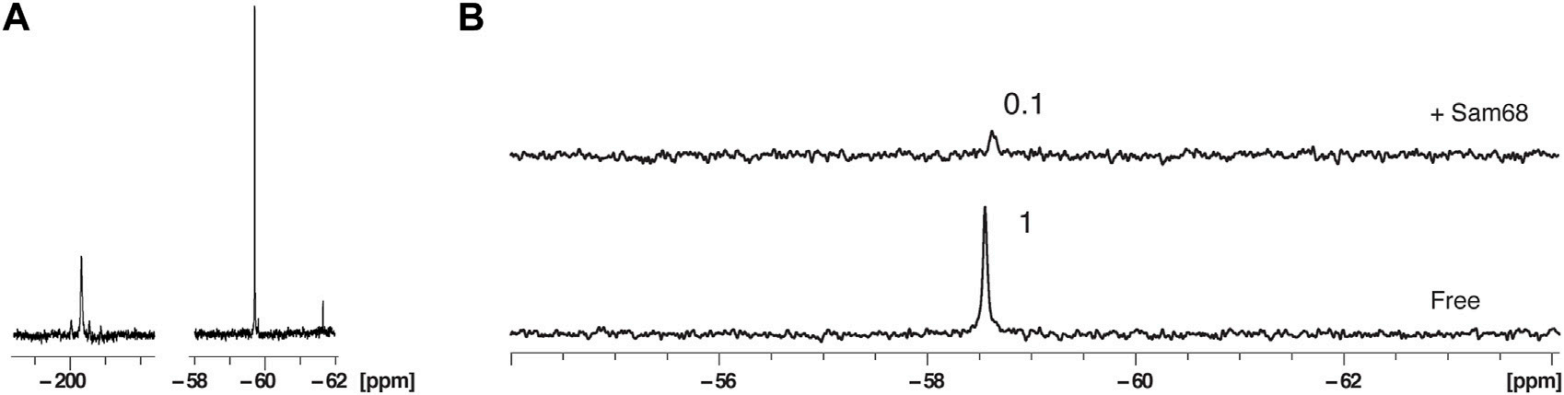
Effect of 2′-OCF3 labelling on ^19^F NMR signal sensitivity and protein binding in the presence of nuclear extract. **(A)** 1D NMR spectra at 303K of fluorinated 2′F-RNA at position A20 at a concentration of 200 μM (left) and fluorinated 2′-OCF3-RNA at position A20 at a concentration of 100 μM (right). The total NMR measurement time per spectrum was 29 min (1,024 scans). **(B)** 1D NMR spectra at 303K of the 2′-OCF3-RNA in the presence of 40% nuclear extract before and after addition of Sam68 at a RNA:protein ratio of 1:2. The total NMR measurement time per spectrum was 7 min (256 scans).

We compared the 1D ^19^F NMR spectra of RNA3 in 40% nuclear extract at a concentration of 100 μM with the 1D spectrum of the 2′-F RNA (RNA2) in 40% nuclear extract at a concentration of 200 μM (total NMR measurement time for each spectrum of 29 min). As expected, the ^19^F NMR signal intensity of the 2′-OCF_3_ RNA3 is significantly higher than the ^19^F intensity of the 2′-F RNA2 even with half the concentration (Figure 5A), demonstrating that a suitable NMR signal can be obtained with shorter measurement times.

We next incubated RNA3 at a concentration of 100 μM in 40% nuclear extract and measured a 1D ^19^F spectrum in the absence or presence of Sam68 at a RNA:protein molar ratio of 1:2 (Figure 5B). The total NMR measurement time per spectrum was 7 min. As for the 2′-F RNA, the addition of Sam68 leads to a strong reduction of the ^19^F signal (PIR of 0.1). However, we do not observe any additional peaks as a result of RNA degradation. The fact that Sam68 induces a stronger reduction of the 2′-OCF_3_ signal (PIR of 0.1) than the 2′-F signal (PIR of 0.6, Figure 3B) could be due to the lower affinity of Sam68 for the 2′-OCF_3_ modified RNA in comparison to the 2′-F RNA (Supplementary Figure S5). In that case, at the concentrations used, only partial saturation of the binding site has occurred for the 2′-OCF_3_ RNA resulting in extensive line broadening (intermediate exchange regime) whereas the binding site is fully occupied for the 2′-F RNA and the signals are broadened due to an increase in correlation time experienced by the ^19^F.

## 4 Discussion

Our results demonstrate that ^19^F-labelling of RNAs is a powerful tool to i) probe the interaction between ssRNAs and one or more proteins at near-nucleotide resolution and in physiological conditions and ii) investigate competition or concurrent binding to the ssRNA. The use of fluorine has the important advantage of facilitating the NMR analysis since fluorine is, in contrast to protons, rarely present in cells, has nearly the same sensitivity as protons (Dolbier, 2009; Gronenborn, 2022) and when incorporated at the 2′position does not affect the thermodynamic stability of the RNA (Kreutz et al., 2005). In this work, the labelling of the RNA at two different nucleotides generates two separate signals, allowing the identification of the RNA motif bound by each protein. Because all nucleotides can be ^19^F-labelled by chemical synthesis, this method would allow for a faster and precise mapping of the RNA motif bound by specific splicing factors, without the need for ^15^N and ^13^C labelling and resonance assignment of the RNA. The approach presented here also demonstrates that the presence of 40% nuclear extract does not affect significantly the ^19^F NMR signals, although it does induce RNA degradation, but this issue can be solved by using 2′-OCF3 labelled RNAs, indicating that it is possible to study protein-ssRNA interactions in physiological conditions. The synthesis of fluorinated phosphoramadites at different positions of the sugar or the base have been reported (Scott et al., 2004; Kreutz et al., 2005; Fauster et al., 2012; Scott and Hennig, 2016; Bao and Xu, 2018, 2020; Becette et al., 2020; Himmelstoss et al., 2020; Li et al., 2020; Nußbaumer et al., 2020; Sudakov et al., 2023). While here we demonstrate the power of ^19^F NMR for protein-ssRNA interaction using modification at the 2′position, it is probable that modifications at other positions of the sugar would also be suitable. However, modifications on the base might affect the affinity of the protein to the RNA since protein-ssRNA interactions mainly occur through the RNA bases (Maris et al., 2005; Valverde et al., 2008; Daubner et al., 2013; Nicastro et al., 2015).

We also demonstrate that using two fluorine signals at different nucleotides of the same RNA allows the identification of concurrent or competitive binding of pairs of proteins. Our study was done at single concentrations of proteins to a final RNA:proteins ratio of 1:2:2, but it would be possible to investigate the binding of individual or pairs of proteins more quantitatively by doing NMR titration experiments with various RNA:protein molar ratios. This would provide additional information, for example, an estimate of the affinity of each protein or pairs of proteins for the RNA. While our study was done with a 21-nucleotide ssRNA and only with pairs of proteins, we believe that similar investigations could be done with longer functional RNAs and more than two proteins added simultaneously, providing a precise mapping of RBPs binding sites and information on their competition or concomitant for ssRNA binding in physiological conditions. Accordingly, it was demonstrated recently that ^19^F NMR analysis can be used to investigate ligand binding to an 86-nucleotide RNA riboswitch containing a single 5-fluorocytidine that was incorporated by splint ligation (Sudakov et al., 2023).

## Data Availability

The raw data supporting the conclusion of this article will be made available by the authors, without undue reservation.
