# Supplementary material for: 2′-19F labelling of ribose in RNAs: a tool to analyse RNA/protein interactions by NMR in physiological conditions
Source: Front Mol Biosci. 2024 Feb 14;11:1325041. doi: 10.3389/fmolb.2024.1325041 (PMC10899400; doi:10.3389/fmolb.2024.1325041)
Supplement: Supplementary file 1 [file Presentation1.pdf]

Supplementary Material

2'-19F labelling of ribose in RNAs, a tool to analyse RNA/Protein interactions by NMR in physiological conditions

Hesna Kara[1,2], Alexander Axer[3], Frederick W. Muskett[1,2], Carlos J. Bueno-Alejo[2,4], Vasileios Paschalis[1,2], Andrea Taladriz-Sender[3], Sumera Tubasum[1,2], Marina Santana Vega[5], Zhengyun Zhao[3], Alasdair W. Clark[5], Andrew J. Hudson[2,4], Ian C. Eperon[1,2], Glenn A. Burley[3]*, Cyril Dominguez[1,2]**

[1]Department of Molecular and Cellular Biology, Henry Wellcome Building, University of Leicester, Lancaster Road, LE1 7HB.

[2]Leicester Institute of Structural & Chemical Biology, Henry Wellcome Building, University of Leicester, Lancaster Road, LE1 7HB.

[3]WestCHEM & Department of Pure & Applied Chemistry, University of Strathclyde, 295 Cathedral Street, Glasgow, G1 1XL, UK.

[4]School of Chemistry, University of Leicester, University Road, Leicester, United Kingdom, LE1 7RH.

[5]Biomedical Engineering Research Division, School of Engineering, University of Glasgow, Rankine Building, Oakfield Avenue, Glasgow, UK.

E-mails: eci@le.ac.uk; glenn.burley@strath.ac.uk; cd180@le.ac.uk

1 Supplementary Data: Chemical synthesis of 2′-*O*-Trifluoromethylated (2′-OCF_3) RNAs

2′-OCF_3-A phosphoramidite was synthesized following a protocol published previously *(Himmelstoss et al, 2020).*

General

All reagents and solvents were obtained from commercial suppliers and were used without further purification unless otherwise stated. Purification was carried out according to standard laboratory methods. Starting materials were purchase from commercial suppliers and used without further purification unless otherwise stated.

Purification of solvents

Dry solvents for reactions were purchased from Sigma-Aldrich and stored under nitrogen. Dichloromethane, chloroform, 2-propanol, hexane, methanol, and ethyl acetate for purification purposes were used as obtained from suppliers, without further purification.

Purification of compounds

Thin layer chromatography was carried out using Merck silica plates coated with fluorescent indicator UV254 and were analyzed under both 254 nm and 375 nm UV light or developed using potassium permanganate or *p*-anisaldehyde solution. Normal phase flash chromatography was carried out using 60Å 40-63 μm silica gel from Fluorochem.

Spectroscopic analysis of products

^{1}H NMR, ^{19}F NMR and ^{31}P NMR spectra were obtained on a Bruker AV 400 at 400 MHz, 162 MHz, 376 MHz, and 162 MHz respectively. DMSO-*d*6 referenced at 2.50 ppm (^{1}H), and C*D*Cl_3 referenced at 7.26 ppm (^{1}H).

Reference

Himmelstoss, M., Erharter, K., Renard, E., Ennifar, E., Kreutz, C. & Micura, R. (2020) 2'-O-Trifluoromethylated RNA - a powerful modification for RNA chemistry and NMR spectroscopy. *Chem Sci*, 11(41), 11322-11330.

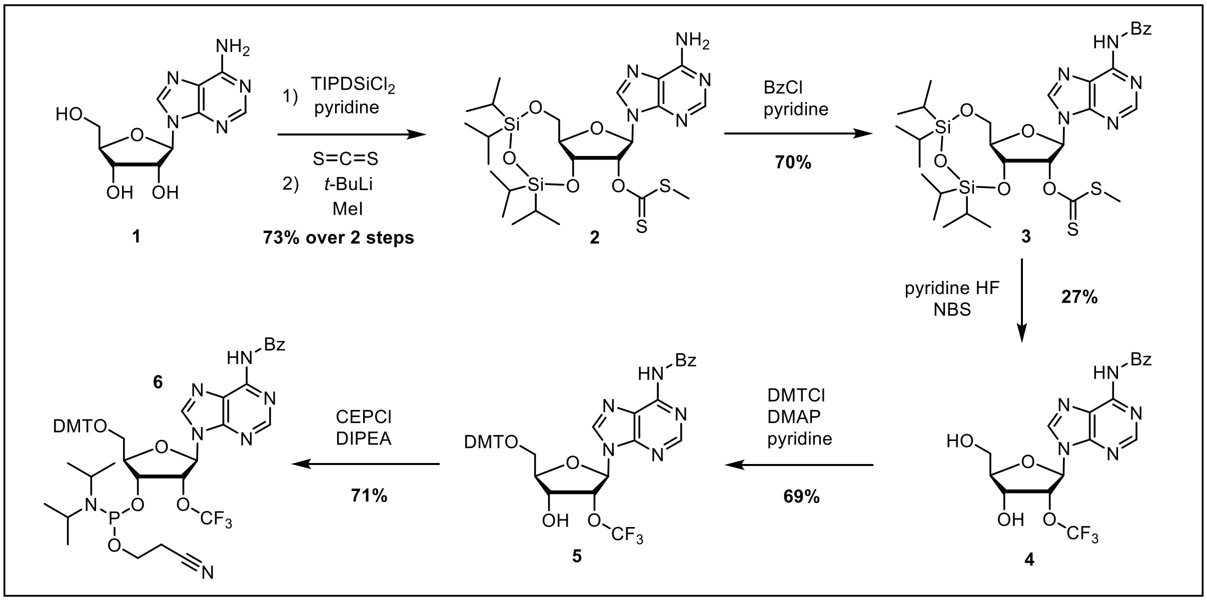

NMR Spectra of compounds 2-6:

^{1}H-NMR (400 MHz, DMSO-d_6) of **2**

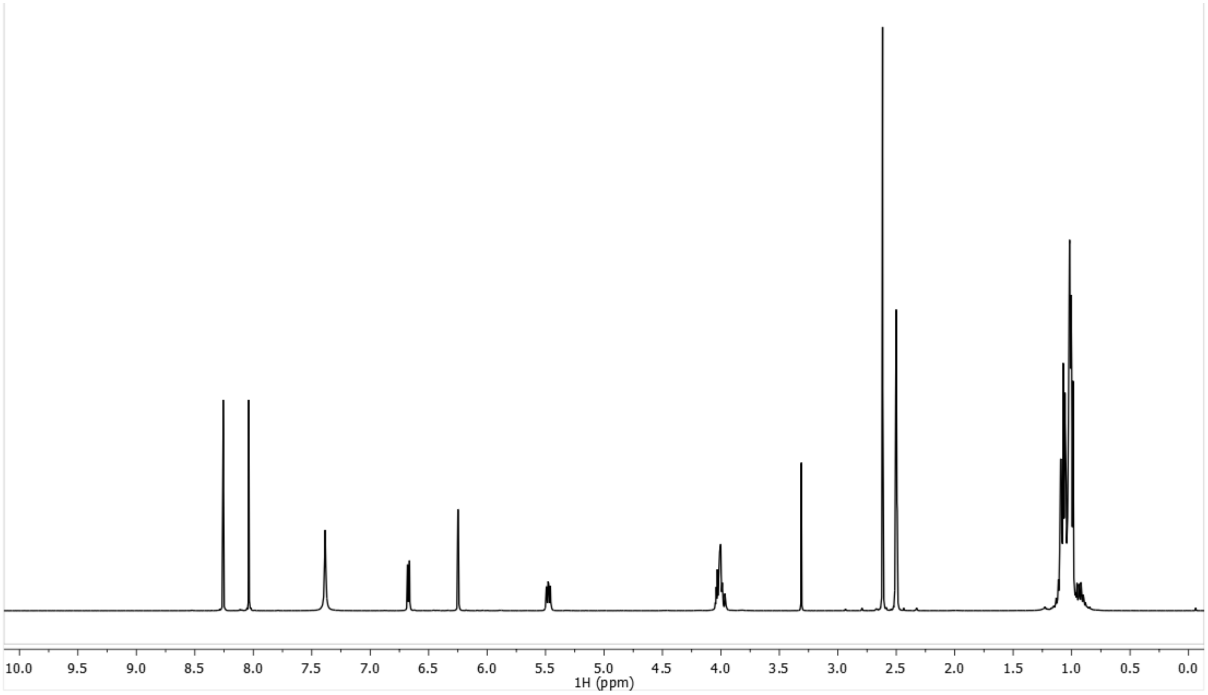

^{1}H-NMR (400 MHz, $CDCl_3$) of **3**

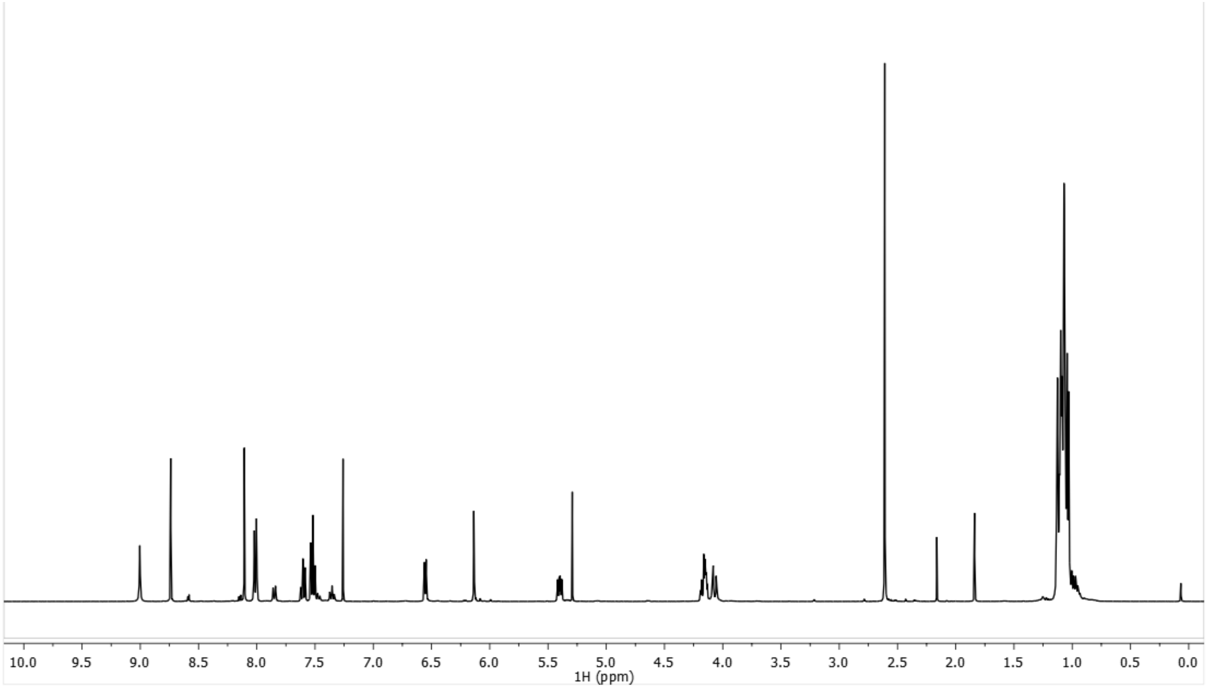

^{1}H-NMR (400 MHz, DMSO-*d*$_6$) of **4**

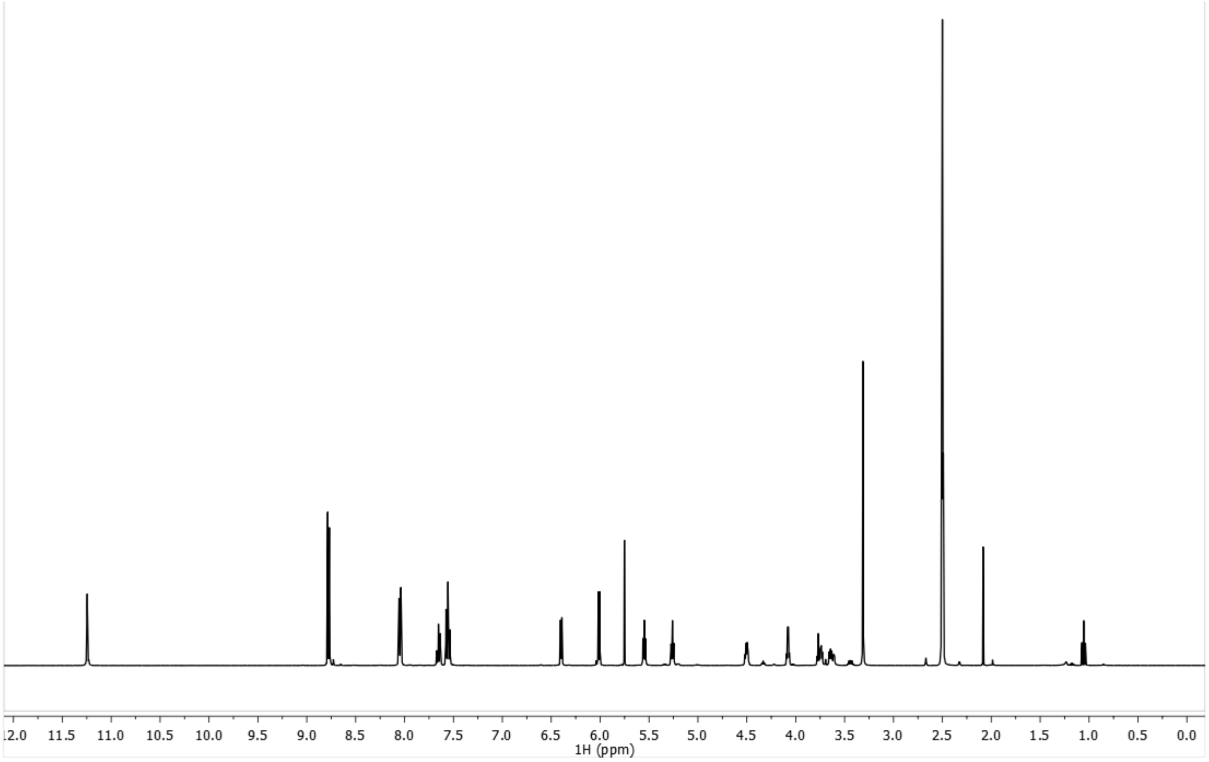

^{19}F-NMR (376 MHz, DMSO-*d*$_6$) of **4**

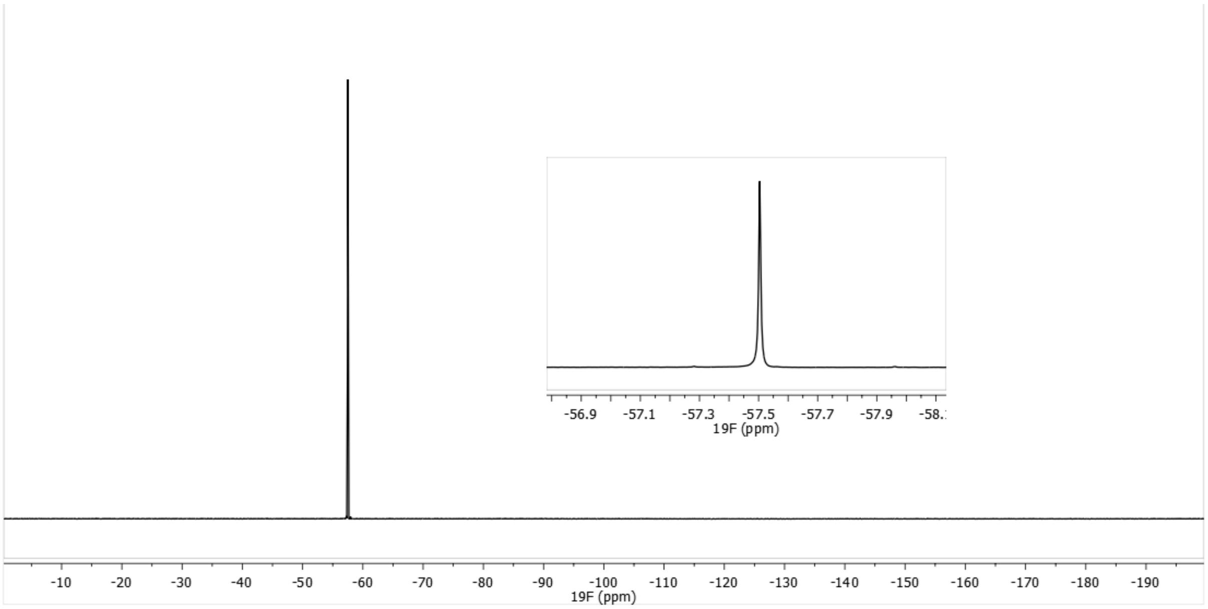

^{1}H-NMR (400 MHz, $CDCl_3$) of **6**

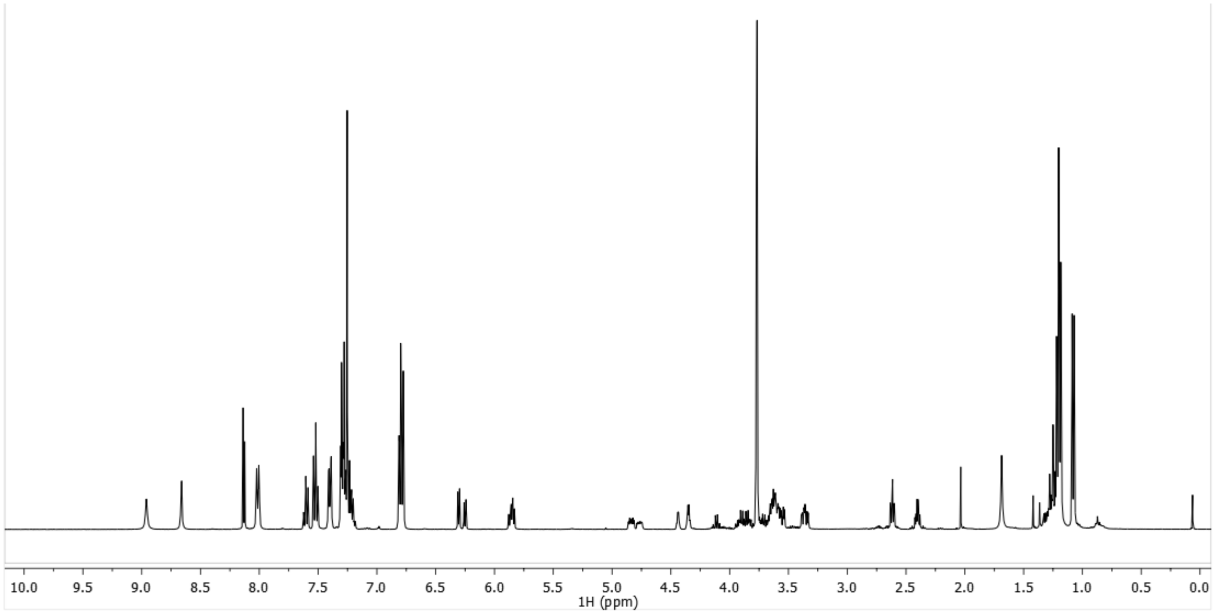

^{19}F-NMR (376 MHz, $CDCl_3$) of **6**

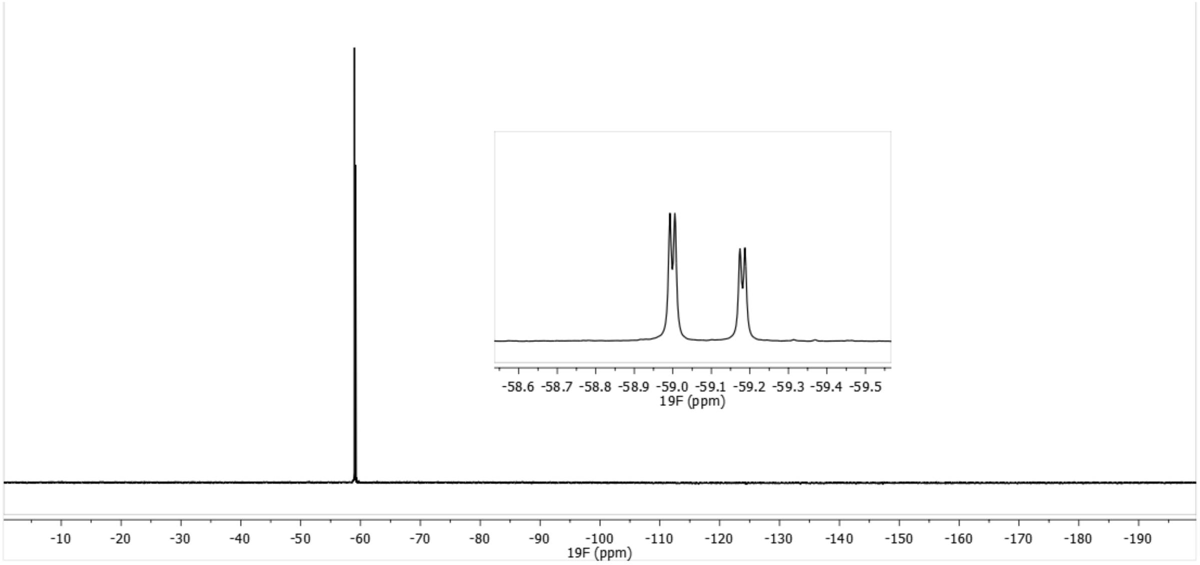

^{31}P-NMR (162 MHz, $CDCl_3$) of **6**

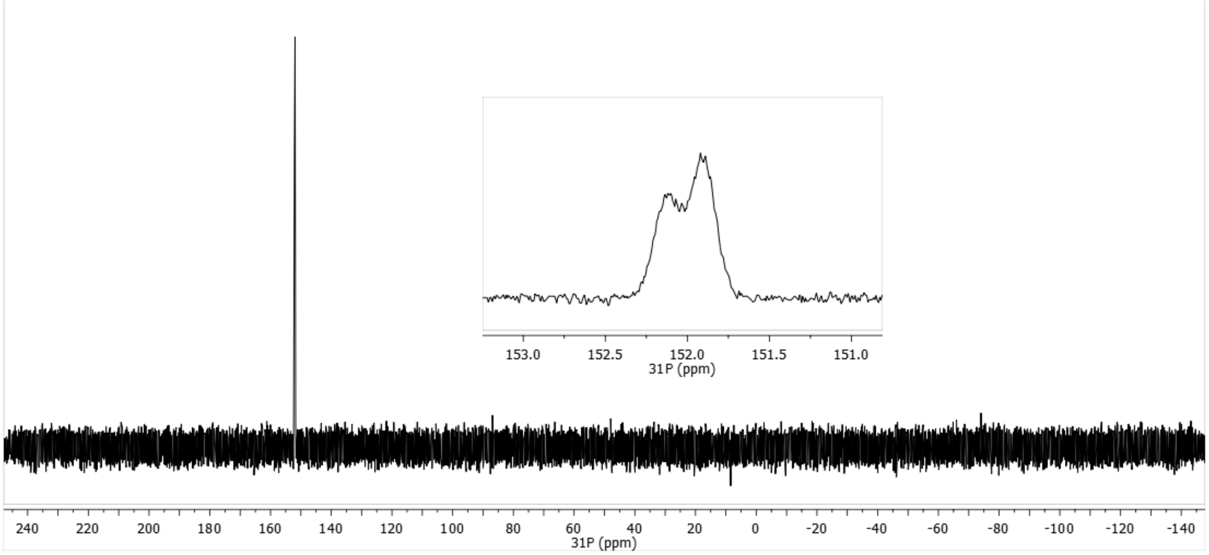

2 Supplementary Figures

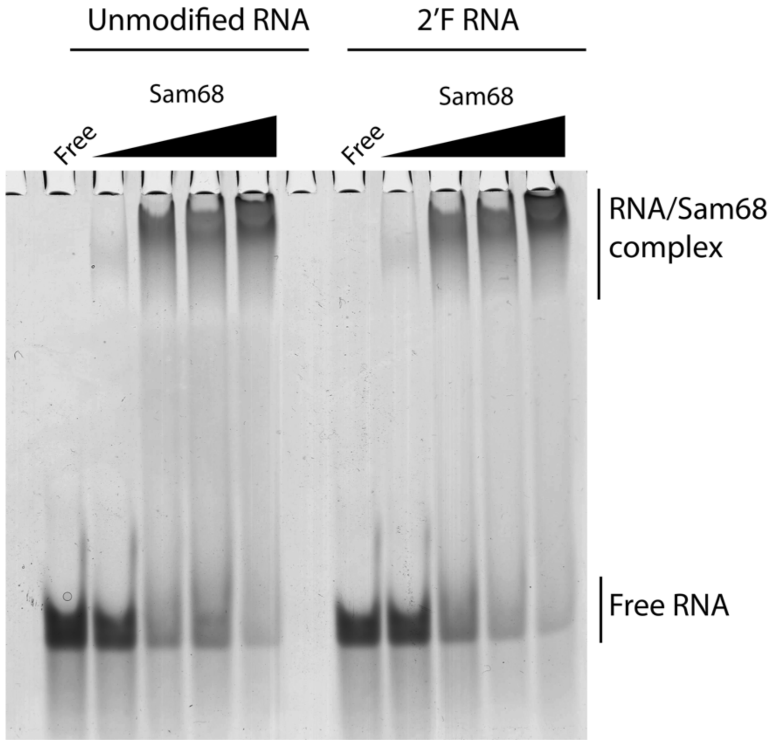

Supplementary Figure S1: Electromobility Shift Assay (EMSA) of unmodified RNA (left) or 2′F RNA2 with and without increasing amount of Sam68. The RNAs (40 µM) and Sam68 (10, 50, 100 and 200 µM) were mixed and incubated for 5 minutes. The samples were then loaded on a 14% native polyacrylamide gel that was run at 160V and at 4° C for approximately 60 minutes, stained with toluidine blue and destained with water.

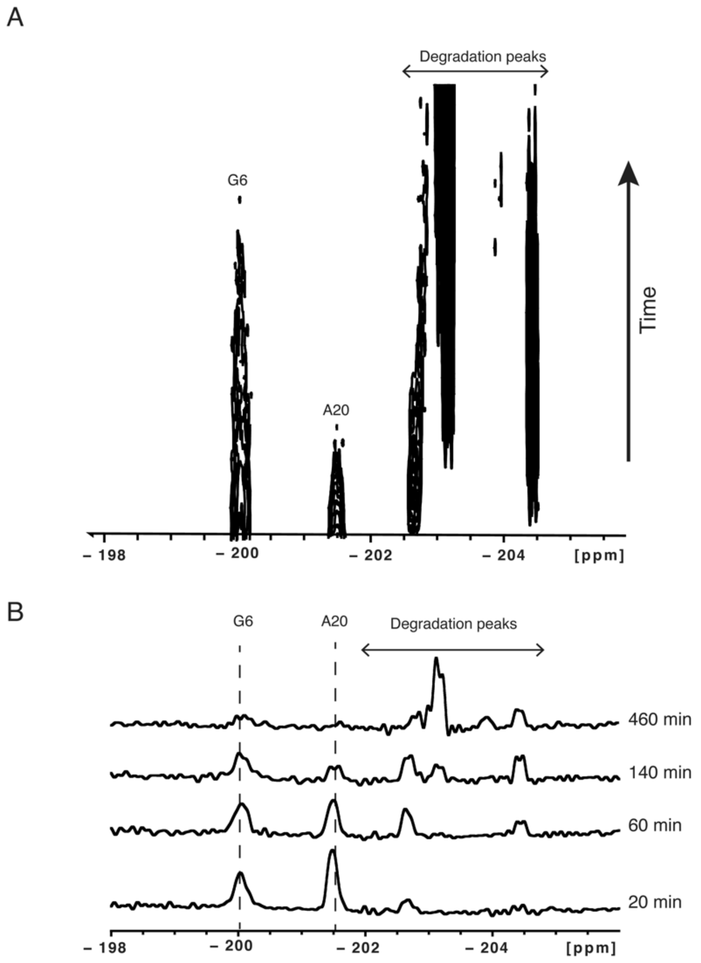

Supplementary Figure S2: ^{19}F 1D NMR of RNA2 in nuclear extract as a function of time. Pseudo-2D traces (A) and 1D ^{19}F spectra (B) of 200µM RNA2 in 40% HeLa nuclear extract as a function of time showing the disappearance of the signals corresponding to G6 (-200.2 ppm) and A20 (-201.5 ppm) and the appearance of degradation peaks between -202.5 and -205 ppm. Spectra were recorded at 303K and the total NMR measurement time per 1D spectrum was 19.5 minutes (1024 scans).

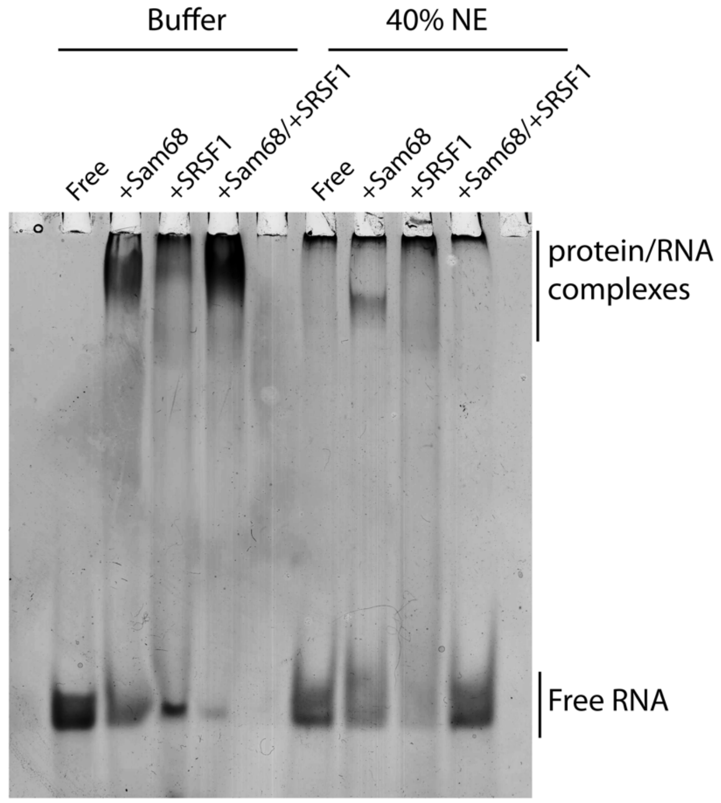

Supplementary Figure S3: Electromobility Shift Assay (EMSA) of RNA2 with and without Sam68 and/or SRSF1 in buffer (left) or in 40% nuclear extract (NE) (right). The RNA (40 µM) and the proteins (80 µM) were mixed and incubated for 5 minutes. The samples were then loaded on a 14% native polyacrylamide gel that was run at 160V for approximately 60 minutes, stained with toluidine blue and destained with water.

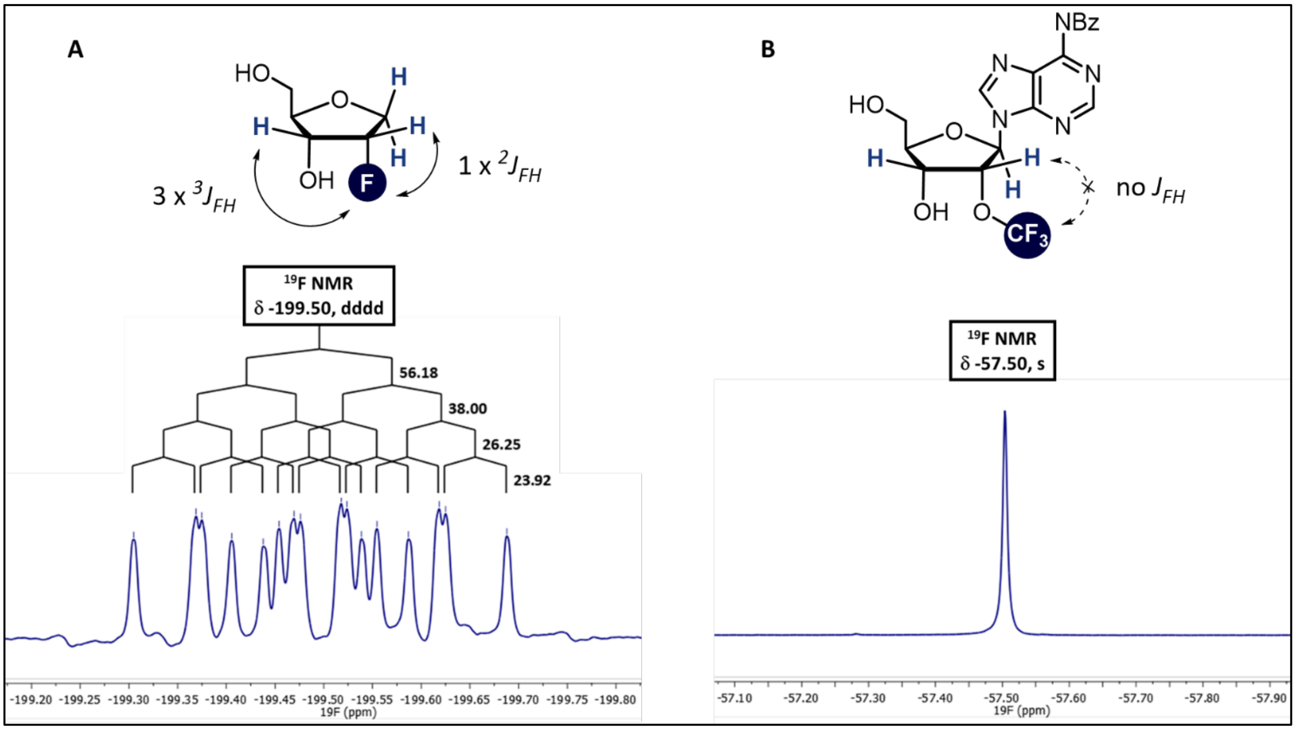

Supplementary Figure S4: 1D ^{19}F NMR spectra of 2'-F-A phosphoramidite in Methanol-d_4 (A) and 2'-OCF$_3$-A phosphoramidite in DMSO-d_6 (B). Spectra were recorded on a Bruker-AVANCE 400MHz at 298K, 32scans, spectra width of 75000Hz.

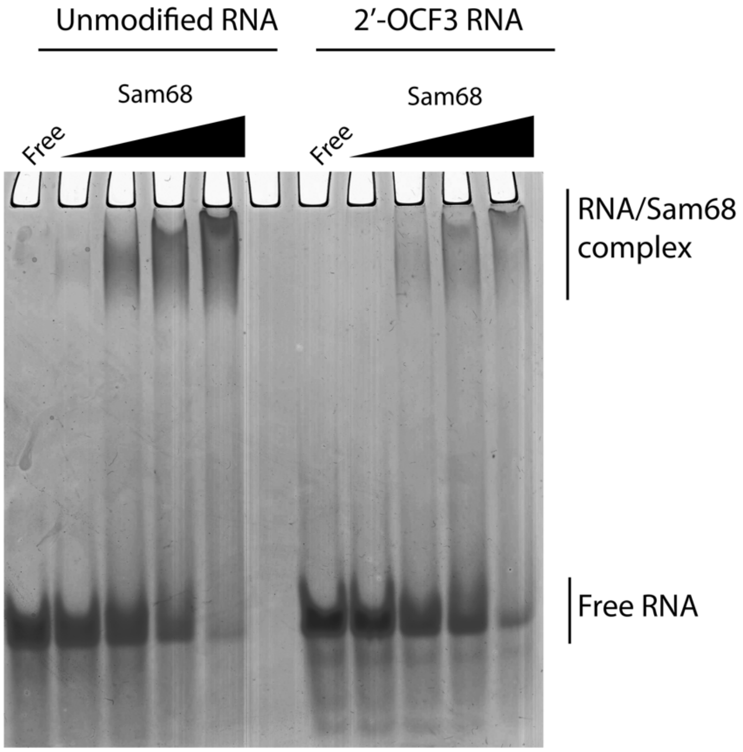

Supplementary Figure S5: Electromobility Shift Assay (EMSA) of unmodified RNA (left) or 2′-OCF3 RNA with and without increasing amount of Sam68. The RNAs (40 µM) and Sam68 (10, 50, 100 and 200 µM) were mixed and incubated for 5 minutes. The samples were then loaded on a 14% native polyacrylamide gel that was run at 160V and at 4° C for approximately 60 minutes, stained with toluidine blue and destained with water.
